# Microplastics in Natural Waters: Occurrence, Risks and Mitigation Strategies

**DOI:** 10.3390/toxics14040296

**Published:** 2026-03-29

**Authors:** Shuwen Zheng, Zhenyu Zhai, Zheming Zhang, Jianxiong Xiang, Jingsi Chen, Zhuorong Du, Xiaoyan Qian

**Affiliations:** 1 Suzhou Medical College, Soochow University, Suzhou 215123, China; zhengshuwen0606@163.com (S.Z.); zhemingzhang2@163.com (Z.Z.); jschen1993@suda.edu.cn (J.C.); 2 The Executive Committee of the Yangtze River Delta Green and Integrated Ecological Development Demonstration Zone, Shanghai 201713, China; zzyhr168@126.com; 3 School of Electrical Engineering, Shanghai University of Electric Power, Shanghai 200090, China; xiangjianxiong0324@163.com; 4 Suzhou Industrial Park Centers for Disease Control and Prevention, Suzhou 215021, China

**Keywords:** microplastics, natural waters, pollution, health risks, control

## Abstract

Microplastics have become a ubiquitous environmental contaminant in natural waters, raising significant concerns regarding aquatic ecosystem health and potential human exposure. A comprehensive synthesis of current knowledge on microplastic pollution in freshwater and marine systems is presented, focusing on sources, distribution patterns, environmental behavior, and associated risks. In freshwater environments, microplastic inputs are closely linked to human activities and land use, with wastewater treatment plant effluent, urban runoff, and agricultural drainage serving as major pathways. In marine systems, microplastics undergo dynamic transport influenced by particle properties, hydrodynamic conditions, and biological interactions such as biofouling and aggregation, leading to widespread distribution from coastal zones to deep sea sediments. Importantly, the role of the freshwater–estuarine–marine continuum is emphasized, highlighting the coupled processes of transport, retention, and remobilisation that govern the spatiotemporal distribution and ultimate fate of microplastics across interconnected aquatic systems. Toxicological effects on aquatic organisms are further examined, particularly immunotoxicity and neurotoxicity, alongside potential human health risks via ingestion, inhalation, and dermal exposure. Attention is drawn to the discrepancy between experimental exposure conditions and environmentally relevant concentrations, which constrains robust risk assessment. Current mitigation strategies, including source reduction, wastewater treatment upgrades, transport interception, and degradation technologies, are critically evaluated in terms of effectiveness and limitations. A clear distinction is made between apparent removal and actual degradation, with further consideration of the environmental implications associated with sludge retention and degradation byproducts. Finally, key research priorities are identified, including the need for standardized detection methods, improved exposure assessment, development of environmentally benign alternatives, and strengthened policy-driven source control. These insights provide a basis for advancing sustainable management strategies for microplastic pollution in natural waters.

## 1. Introduction

Over recent decades, plastic has increasingly replaced traditional materials due to its cost-effectiveness, reusability and durability [1]. Projections based on prevailing consumption patterns and waste plastic management practices indicate that approximately 12 billion metric tons of plastic waste will be disposed of in landfills or released into the natural environment by 2050 [2]. Furthermore, since the onset of the COVID-19 pandemic, the widespread use and improper disposal of masks have contributed significantly to plastic pollution [3]. Based on estimates of the global usage of disposable medical and personal protective equipment during the pandemic, the amount of plastic waste generated worldwide may have reached up to 1.6 million tons per day at the peak of the outbreak [4]. Owing to high plastic consumption and low recycling rates, the total amount of plastics in the environment continues to increase annually. As these plastic materials accumulate in the environment, they undergo fragmentation and degradation, leading to the formation of microplastics that are widely distributed across environmental compartments [5].

Microplastics are generally defined as plastic particles smaller than 5 mm, a concept first formally introduced by Thompson et al. in 2004 [6]. They have been widely detected across multiple environmental compartments, including the atmosphere, soils, aquatic systems, and biota [7]. Based on their origin, microplastics are typically classified into primary and secondary types. Primary microplastics are intentionally manufactured for commercial or industrial use, such as the plastic exfoliating particles used in facial cleansers and other cosmetics including scrubs and toothpaste [8]. The content of primary microplastics in these products can reach up to 50,391 particles per gram, with a single use potentially releasing up to 229,000 microbeads into domestic wastewater [9]. Secondary microplastics refer to those generated from the fragmentation of larger plastic items through physical and chemical processes such as abrasion and weathering, or via biological degradation processes [10]. Therefore, understanding the sources and characteristics of microplastics requires consideration of the types of macroplastics, transformation conditions, and distribution patterns.

In recent years, microplastics have emerged as a prominent research focus, with numerous review articles published on the subject. However, the majority of these reviews on microplastics in natural water bodies predominantly focus on the contamination of marine environments by microplastics [11,12,13,14,15], while relatively few studies have specifically addressed freshwater ecosystems [16,17,18,19,20]. Systematic comparative analyses of source differences, contamination characteristics, transport and transformation mechanisms, and fate models between freshwater and marine systems remain relatively scarce, highlighting the need for a more holistic perspective. Moreover, most existing reviews have evaluated the impacts of microplastics in aquatic environments on marine organisms [21,22]. In comparison, the potential implications of microplastic exposure for human health have begun to receive increasing attention. However, several uncertainties remain, including the limited understanding of environmentally relevant exposure levels and the discrepancy between concentrations used in experimental studies and those encountered under real environmental conditions. These inconsistencies hinder the establishment of robust dose–response relationships for risk assessment. Given the scope of this review, which mainly focuses on the microplastics in aquatic environments, the discussion of human health impacts in this paper primarily summarizes mechanistic insights derived from experimental toxicology studies. Existing reviews consistently acknowledge that microplastics are ubiquitously distributed in the environment, where they can act as vectors for the adsorption of organic pollutants and heavy metals, potentially leading to complex combined toxic effects with potential risks to ecosystems and human health. Nevertheless, critical knowledge gaps persist regarding their transport mechanisms and long-term toxicological impacts, underscoring the necessity for further research and the refinement of regulatory measures [7,23,24].

In addition to microplastics, nanoplastics, typically ranging from approximately 1 to 100 nm, have also emerged as a topic of growing concern [25]. A recent systematic synthesis of human biomonitoring studies comparing micro- and nanoplastics across multiple biological matrices indicates that, although both particle types have been detected in various human tissues and fluids, current evidence on nanoplastics remains highly limited due to analytical constraints and the scarcity of quantitative data [26]. Therefore, this review primarily focuses on microplastics, while nanoplastics are addressed only when relevant to recent research advances and toxicological mechanisms associated with microplastics.

This review examines the sources and hazards of microplastics and synthesizes analyses by domestic and international scholars regarding the current state of microplastic pollution and corresponding prevention and control strategies. It is noteworthy that certain advanced technologies, such as Membrane Bioreactors (MBRs), can achieve a removal efficiency of up to 99.9% for microplastics in wastewater [27], thereby providing an effective technological option for pollution mitigation. Overall, this review aims to provide a scientific basis for developing targeted pollution prevention and control strategies and promoting the sustainable management of natural aquatic environments, ultimately contributing to the protection of aquatic ecosystem integrity and human health.

## 2. Bibliometric Analysis

**Figure 1:**
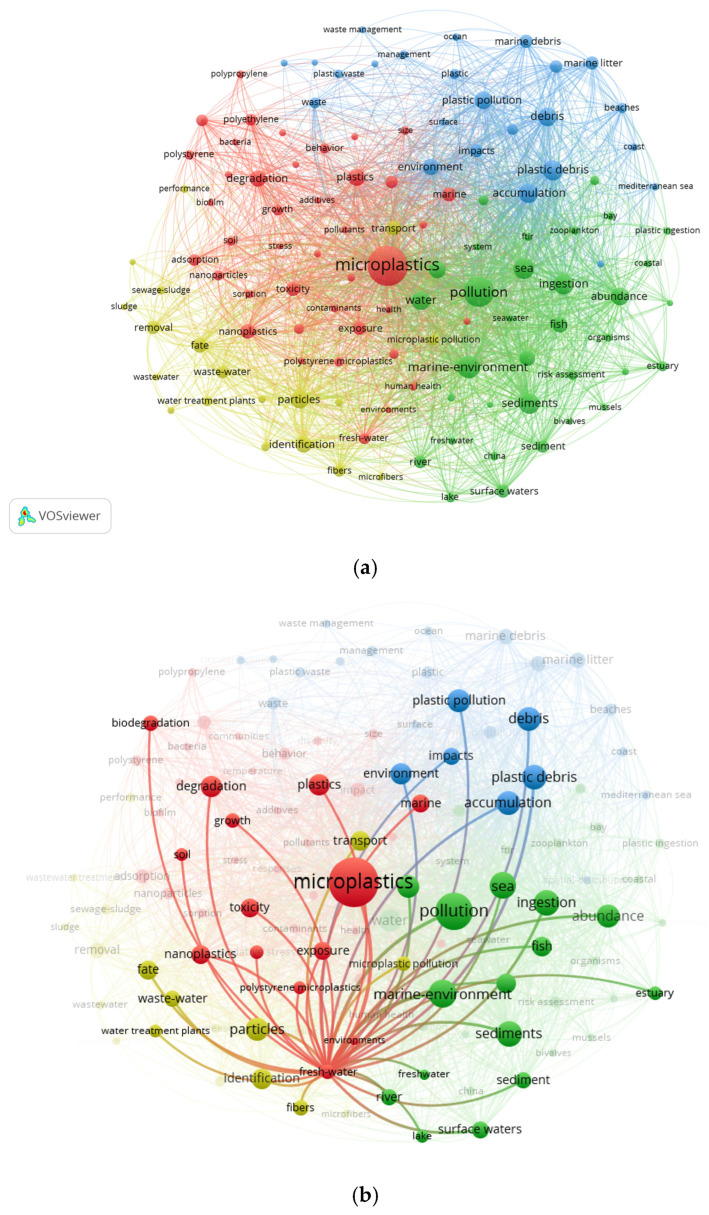

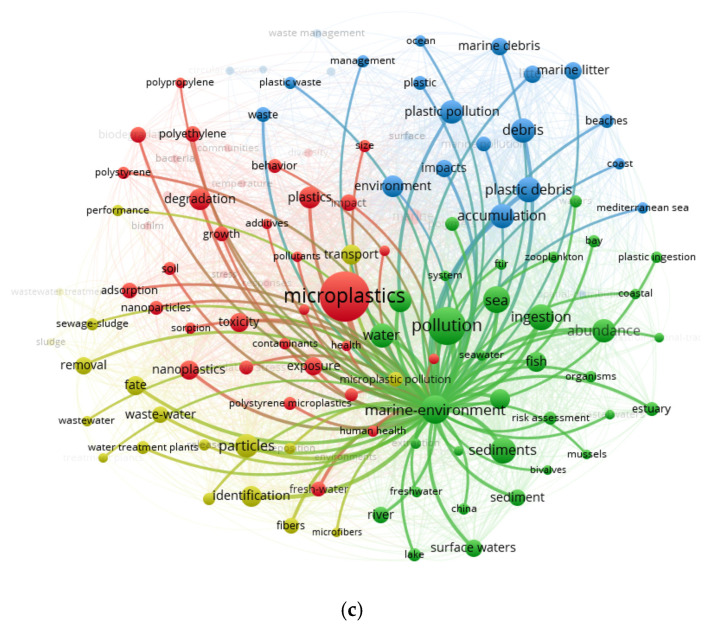
Bibliometric analysis of microplastics in natural waters using Web of Science: (**a**) microplastics (general), (**b**) freshwater, and (**c**) marine environment.

We collected the data on “microplastics in natural waters” from published scientific articles from January 2021 to January 2026 (including January 2026) using the Web of Science Core Collection database. The keywords were chosen as (TS=(microplastics OR plastic debris OR microbeads OR plastic OR plastic contamination OR microlitter OR plastic fragment OR nanoplastics OR nanopolymer)) AND TS=(marine OR ocean OR sea OR coastal OR freshwater OR inlandwater OR river OR wastewater OR lake OR creek OR sewage OR pond OR reservoir OR stream)) AND TS=(source OR occurrence OR distribution OR pollution OR contamin OR hazard OR risk OR toxic OR control OR remedi OR management). Studies were included if they met the following criteria: (i) the publication type was restricted to research articles and review papers to ensure sufficient methodological detail and scientific rigor; (ii) no language restrictions were applied; and (iii) the study focused on microplastics in natural waters. Conference abstracts, editorial materials, news items, and book chapters were excluded due to their limited academic content. We extracted metadata including publication year, journal, authorship, country of origin, and keywords for quantitative analysis of research trends and thematic distributions. All subfigures in Figure 1 were generated using VOSviewer (version 1.6.20). A keyword threshold of 120 was applied to filter out low-frequency words, and Max.line was set at 2000. The resulting keyword co-occurrence network was visualized using the software’s clustering algorithm to identify major research themes and their relationships across general, freshwater, and marine microplastics studies.

As shown in Figure 1, the bibliometric mapping identifies four major research clusters in studies of microplastics in natural waters over the past five years:

1. Toxicological impacts, particularly of nanoplastics, including oxidative stress, cellular damage, bioaccumulation, and potential human health risks.
2. Environmental occurrence and distribution, encompassing the abundance, spatial patterns, and current pollution status of microplastics in marine and freshwater environments, sediments, and aquatic organisms.
3. Ecological dynamics and ecosystem impacts, highlighting the role of aquatic ecosystems as major sinks for microplastics, their transport and transformation processes, structural and functional consequences for ecosystems, and ecological risks associated with food chain transfer via plastic ingestion.
4. Analytical methods and mitigation strategies, covering detection and identification techniques, degradation mechanisms, removal technologies, and wastewater treatment applications, with research increasingly emphasizing sustainable management approaches.

Emerging subtopics, such as nanoplastics, have attracted increasing attention, while research on marine environments primarily addresses global ecological impacts and risk assessment, with studies focusing on marine debris abundance, hazards associated with biological ingestion, and global circulation models. In contrast, research in freshwater systems remains comparatively limited, emphasizing sources, transport pathways, and control technologies within freshwater environments. These two research streams are gradually converging, and greater attention should be directed toward the microplastic continuum from freshwater through estuarine to marine systems. Based on bibliometric findings, future efforts should prioritize investigations into the environmental behavior and toxicological mechanisms of nanoplastics, as well as facilitate the translation of efficient, low-cost removal technologies from laboratory research to practical applications.

## 3. Sources and Pollution Status

Since wastewater treatment cannot completely remove microplastics before discharge, a significant amount enters water bodies via treated effluent and sludge [28]. Additionally, the transport of microplastics through water movement in the atmosphere and soils can facilitate their transfer. Given the greater human reliance on freshwater and the differing usage patterns between freshwater and marine system, the characteristics of microplastics in these two environments vary. Accordingly, this section discusses the sources and pollution status of microplastics in aquatic environments by separately addressing freshwater and marine systems.

### 3.1. In Freshwater

As a commonly utilized water source in human production and daily activities, freshwater bodies are characterized by a significant presence of microplastics, most of which originate from human activities. A review and quality assessment of data on microplastics in freshwater and drinking water indicates that microplastics in water samples primarily exist as fragments, fibers, films, foams, and pellets [29]. The pathways through which microplastics enter freshwater environments are complex and include: atmospheric deposition from urban and industrial dust, construction activities, waste incineration, and traffic emissions; degradation of equipment used in fisheries, agriculture, and aquaculture; effluent from wastewater treatment plants; terrestrial runoff and stormwater that wash plastic debris from land surfaces into rivers and lakes; and fibers shed from daily-use products and industrial materials, which enter freshwater systems indirectly via sewage networks [19]. Through these diverse and continuous input pathways, microplastics accumulate in freshwater systems, leading to widespread pollution globally.

From a source-type perspective, microplastic inputs can broadly be categorized into point sources and diffuse sources [30]. Current research generally recognizes that the effluent from wastewater treatment plants (WWTPs) and various runoff are the main sources of microplastics in freshwater systems [31]. WWTPs represent a typical point source of microplastic pollution, discharging substantial quantities of microplastics derived from synthetic textile fibers, personal care products, and other sources into aquatic environments [32,33]. Urban stormwater runoff serves as a significant pathway for transporting tire and road wear particles, as well as fragmented plastic debris from anthropogenic waste, into adjacent rivers and lakes [34,35]. Additional sources of microplastics, such as atmospheric deposition and degradation of plastic materials utilized in agricultural and aquaculture practices, exhibit contamination levels that are concurrently influenced by meteorological patterns, including diverse microplastic transport pathways and mass flows [36]. Moreover, urban environments are predominantly contaminated by wastewater discharge and surface runoff from built-up areas and roadways, whereas microplastic pollution in rural regions primarily originates from the use and fragmentation of agricultural plastic films and farming implements [28,37]. This distinction highlights the importance of considering both land-use patterns and source types when evaluating freshwater microplastic pollution.

Table 1 summarizes the monitoring data of microplastics in freshwater bodies across different countries and regions in the world. It is important to emphasize that the uncertainty and heterogeneity of these studies must be taken into account. These differences do not merely reflect the actual environmental conditions, and a small portion of them stems from methodological disparities. Firstly, there are differences in sampling techniques, such as whether surface water or sediment is collected, and the choice of filter mesh sizes. Secondly, there are variations in analytical methods, and the use of different units for reporting microplastic concentrations across studies further complicates direct comparisons.

As illustrated in Table 1, the distribution of microplastics in aquatic environments demonstrates global, heterogeneous, and complex characteristics. Findings indicate that microplastics are ubiquitously present in various freshwater bodies worldwide. High abundances of microplastics have been detected in economically active urban rivers, such as the Old Brahmaputra River in Bangladesh, the lower reaches of China’s Yangtze and Yellow Rivers, as well as in inland lakes. Even remote high-altitude lakes with minimal anthropogenic disturbance, such as Nepal’s Tilicho Lake, have shown detectable levels of microplastics. The spatial distribution of microplastics in freshwater systems exhibits marked heterogeneity: aquatic systems in densely populated regions display significantly higher microplastic abundances than those in remote natural areas. Due to hydrodynamic transport and sedimentation processes, downstream river sections and estuarine confluence zones serve as major accumulation hotspots for microplastics. A recent study also identified microplastic pollution in European groundwater [49]. Furthermore, an analysis of the material composition reveals that the primary polymer components identified in freshwater microplastics are polyethylene (PE), polypropylene (PP), polystyrene (PS), and polyethylene terephthalate (PET), accounting for approximately 70% of the total [18].

The aforementioned discussion further underscores the necessity of establishing standardized analytical methodologies: variations in particle size screening thresholds directly influence the quantification of microplastic abundance, while disparities in identification accuracy and detection limits among different analytical methods persist. Unified sampling, sample pretreatment, and analytical standards constitute a pivotal prerequisite for conducting accurate assessments of global freshwater microplastic pollution levels. Future research will greatly benefit from the development and adoption of standardized protocols to minimize methodological variability and enable more reliable global assessments.

### 3.2. In Marine Environment

Research on marine microplastics dates back to 1972, when Carpenter et al. published their seminal paper on the subject [50]. In marine environments, microplastics typically exist as particles, fragments, or fibers composed of various polymers. Denser particles, such as those made of PVC, polyester, and polyamide, tend to sink and accumulate in seafloor sediments, whereas lighter particles like polyethylene, polypropylene, and polystyrene are more likely to float on the sea surface [51].

However, the environmental behavior of microplastics cannot be fully explained by polymer density alone. In marine environments, processes such as biofouling, aggregation, and fragmentation continuously modify the physical characteristics of microplastic particles over time. Plastic debris with a density higher than that of water will sink directly; however, low-density microplastics may undergo biofouling due to microbial colonization on their surfaces. This process increases the particle density and can cause otherwise buoyant polymers such as polyethylene and polypropylene to sink [52]. Furthermore, microplastics can undergo aggregation with mineral particles and marine snow, forming larger composite aggregates that facilitate their vertical transport to deeper water layers and sediments [53,54]. The fragmentation processes induced by ultraviolet radiation and mechanical abrasion further alter particle size distributions, resulting in an increased abundance of polymeric particles with decreasing dimensions [55]. Consequently, these changes significantly influence transport dynamics and environmental dispersion. As a result, the vertical distribution of microplastics in marine systems represents a dynamic process, and interpretations based solely on intrinsic polymer density may oversimplify the long-term environmental fate of these particles.

Land-based sources account for 75–90% of microplastics in marine debris, with marine-based sources contributing only 10–25% [56]. It should be noted that marine debris typically refers to plastic waste observed directly on beaches or at the sea surface, such as abandoned fishing gear or shoreline litter, which reflects the composition of macroplastic sources [57]. Land-based sources, such as urban runoff and river discharge, serve as the major contributor to marine microplastic pollution. These inputs, combined with direct human emissions, agricultural runoff, atmospheric transport, and hydrological processes, form a complex microplastic circulation cycle [58]. Current research indicates that microplastic pollution is particularly severe in coastal waters, largely due to large-scale anthropogenic emissions. Microplastics from coastal areas enter the ocean through various pathways, exacerbated by the development of industries such as coastal fisheries and maritime transport. A study estimated that in 2010 alone, between 4.8 and 12.7 million metric tons of plastic waste entered the ocean from coastal cities globally [59]. This staggering amount not only poses direct hazards as macro-debris but also leads to secondary pollution: when these plastics undergo abrasion and weathering in seawater, they release vast quantities of microplastics. Marine-based sources primarily include seaside tourism, commercial fishing, marine vessels, and offshore industries [60]. With the expansion of mariculture, studies show that in some aquaculture zones, 83% to 99% of microplastic particles originate from the farms themselves [61], making these operations a major pollution source in surrounding coastal waters.

Microplastics are distributed throughout the world’s oceans in substantial quantities, found on beaches, in the water column, and in sediments—with evidence of their presence extending even to the Arctic Ocean [23].

**Coastal Waters:** Coastal waters, serving as the primary sink for land-based microplastics and a core area for human marine activities, represent the most concentrated zones of global microplastic pollution. Studies show that microplastic concentrations in beach sediments can reach as high as 13,418.86 ± 28,787.99 particles/m^2^—approximately 100 times higher than in seafloor sediments and 1000 times higher than in surface seawater. These concentrations closely match the pollution characteristics of nearby municipal sewage and industrial discharges [62]. Furthermore, coastal pollution levels exhibit a positive correlation with population density and regional industrialization, indicating diverse and significant sources of microplastics in these areas [63].

**Open Ocean:** Although remote from direct human activity, the open ocean has become an important zone for the long-range transport and accumulation of microplastics through ocean currents, atmospheric deposition, and shipping activities. The great ocean garbage patches are typical manifestations of such pollution [64]. Recent studies indicate that polyethylene and polypropylene dominate the microplastic composition in open-ocean samples, originating largely from surface subsidence, with vertical transport serving as a key dispersal pathway [65].

**Polar and Remote Marine Areas:** Polar seas, including the Arctic and Antarctic, as well as remote islands, once regarded as the “pure lands” of the global ecology, have been shown in recent years to be contaminated by microplastics [66]. In polar regions, microplastics are mainly delivered via long-range transport through ocean currents and atmospheric circulation from human-active areas. Local sources such as research stations, tourism, fishing vessels, and atmospheric deposition via snowfall and dry deposition also contribute. Compared to the Arctic, research on microplastics in the Antarctic remains relatively limited [67].

**Deep-Sea Environments:** As a critical component of marine ecosystems, the deep sea (depths > 1000 m) acts as a hidden accumulation zone for microplastic pollution. Sediments from the Mariana Trench, for example, contain microplastic abundances ranging from 200 to 2200 particles per liter, significantly higher than in most other deep-sea sediments [68]. These results reveal that anthropogenic plastic pollution has infiltrated the most remote and deepest regions of the planet, substantially enhancing our understanding of contamination in deep-sea ecosystems.

### 3.3. Freshwater–Estuarine–Marine Continuum

Microplastic pollution is evolving into a continuum that intimately interconnects terrestrial, freshwater, estuarine, and marine systems. Plastic debris from terrestrial activities enters freshwater systems through multiple pathways, including discharges from wastewater treatment plants, stormwater runoff, agricultural drainage, and atmospheric deposition [58]. Subsequently, rivers serve as key conduits for transporting microplastics from inland areas to estuarine and coastal marine environments [69]. This transport is not unidirectional but is dynamically regulated by coupled processes of advection, retention, and episodic remobilisation along the river continuum. In riverine systems, microplastics are influenced by suspension, sedimentation, and resuspension processes. They are not only transported downstream but may also be deposited and temporarily stored in riverbed sediments [70]. These retained particles can be remobilised during high flow events, such as floods, or hydrodynamic disturbances, thereby re-entering the water column and contributing to pulsed downstream transport [71].

Estuarine and coastal wetlands serve as critical transition zones (CTZs) controlling the transport of microplastics from freshwater systems to marine environments [72]. Hydrodynamic processes such as tidal mixing and the flocculation and settling of sediments influence the retention, transformation, and redistribution of particulate matter within these environments [73,74]. In particular, estuarine trapping arises from the longitudinal convergence of particle transport driven by coupled hydrodynamic processes, including gravitational circulation induced by salinity gradients, tidal pumping and asymmetry, and particle aggregation [75,76,77]. These processes give rise to the formation of estuarine turbidity maxima, which act as hotspots for microplastic accumulation and prolonged residence time.

In summary, microplastics transported by rivers may temporarily accumulate in sediments, wetlands, and estuaries. However, these retained particles are not permanently immobilized; climate- and tidal-driven mixing, as well as sediment resuspension, can remobilize them and facilitate downstream transport. Thus, retention and remobilisation operate as coupled processes that regulate the timing, magnitude, and intermittency of microplastic fluxes across system boundaries. Consequently, estuaries function not only as sinks for microplastics but also as dynamic transitional zones that modulate the flux of microplastics from inland waters to coastal and marine environments. Depending on hydrodynamic conditions, estuaries may either attenuate downstream transport through trapping or act as secondary sources that release previously stored microplastics to coastal waters. Meanwhile, a portion of microplastics is directly conveyed to coastal waters through riverine discharge and tidal action.

Once released into the marine environment, microplastics can undergo rapid and extensive dispersion through processes such as ocean currents and wind-driven transport. Over time, these particles may undergo vertical transport, ultimately settling into deep-sea ecosystems [78]. Deep-sea sediments may function as a major sink for marine microplastics. However, similar to upstream systems, marine sediments can also act as reservoirs subject to physical such as bottom currents and storms, and biological remobilisation such as bioturbation, further contributing to their redistribution [79,80,81]. In addition, biomagnification and bioaccumulation constitute another significant pathway for the redistribution of microplastics within aquatic systems [82]. Overall, the interplay among transport, retention, trapping, and remobilisation processes across the freshwater–estuarine–marine continuum governs the spatiotemporal distribution and ultimate fate of microplastics, underscoring the need for an integrated, process-based perspective.

## 4. Health Risks

### 4.1. Ecotoxicity to Aquatic Organisms

As a ubiquitous pollutant, microplastics have permeated all levels of aquatic ecosystems, thereby posing significant threats to the survival and development of aquatic organisms. A recent meta-analysis indicates that microplastics have been identified in diverse aquatic species, including foraminifera, fish, and crabs, with studies conducted across at least 57 countries and regions. Among these, fish represent the most extensively studied group to date [83]. Microplastics can enter aquatic organisms through multiple pathways, influenced by the species, developmental stage, and physiological traits of the organism, as well as the size and morphology of the microplastics. For example, during early developmental stages, fish can ingest microplastics through feeding, gill adsorption, and epidermal adherence [84]. Quantitative research further confirms the main entry routes: the abundance of microplastics in combined gill and gastrointestinal tract samples (15.61 MPs/individual) was 68.8% higher than in gastrointestinal-only samples (9.25 MPs/individual), highlighting that both the gills and digestive tract serve as critical dual pathways for microplastic ingestion in fish [83].

**Figure 2:**
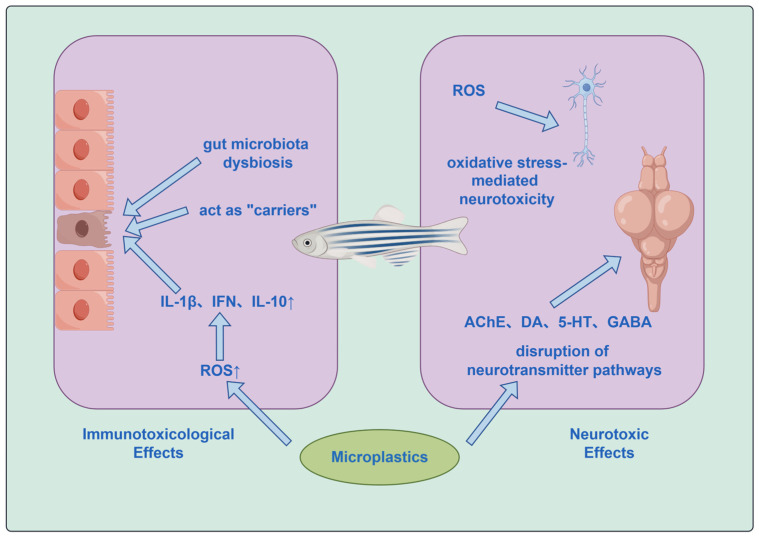
Immunotoxic and Neurotoxic Effects of Microplastics on Aquatic Organisms.

Once inside aquatic organisms, microplastics can cause damage to biological tissues through both physical interference and chemical effects, thereby revealing latent toxicity [85]. These two mechanisms act synergistically and mutually reinforce each other, collectively disrupting the normal physiological metabolism of aquatic organisms, ultimately impairing their growth, development, reproduction, and survival. As shown in Figure 2, current research has confirmed that the toxic effects of microplastics can impact multiple physiological systems in aquatic organisms, including immunity and neural function.

#### 4.1.1. Immunotoxicological Effects

Once inside the organism, microplastics can affect vital immune organs, alter the expression levels of immune cytokines and related genes, and disrupt immune homeostasis [86]. The intestine, as the primary digestive organ, is responsible for nutrient absorption and endocrine and immune functions [87]. Research indicates that the ingestion of microplastics by fish ranges from 1 to 20 particles, with polyethylene and polypropylene being the predominant polymers involved. Following accumulation in the intestinal tract, microplastics induce a significant increase in the production of reactive oxygen species (ROS), which subsequently upregulates the transcription and expression of pro-inflammatory cytokines such as IL-1β, IFN, and IL-10, positively correlating with microplastic concentration. Concurrently, compensatory elevation in the expression of antioxidant enzymes (CAT and SOD) occurs. Cytokine-dependent signaling pathways mediate the infiltration of intestinal immune cells and inflammatory responses, while the anti-inflammatory cytokine IL-10 is simultaneously upregulated, forming an inflammatory feedback regulation loop. This pathway represents the core mechanism underlying microplastic-induced intestinal immunotoxicity in fish [88]. Simultaneously, the gut microbiota interacts with the intestinal immune system to further regulate immune function [89]. A review indicates that gut microbiota dysbiosis induces dysregulated expression of key metabolites, such as vitamin B12, orotic acid, and arachidonic acid, while microbiota dysbiosis-mediated metabolic disturbances further exacerbate intestinal oxidative stress and tissue injury, thereby establishing a vicious cycle [90]. The dysbiosis of gut microbiota may constitute a novel mechanism by which microplastics cause or exacerbate intestinal toxicity in fish [91].

Furthermore, microplastics act as “carriers” that significantly amplify the toxicity of coexisting pollutants, resulting in synergistic toxic effects [92], with notable adverse impacts on the immune system.

#### 4.1.2. Neurotoxic Effects

Microplastics exert neurotoxic effects on fish through multiple pathways, with the core mechanism involving interference in neurotransmitter pathways. A review indicates that the majority of studies employ polystyrene (PS) as a single exposure material. Furthermore, nanoplastics demonstrate an enhanced ability to cross biological barriers, with smaller particle sizes correlating with increased toxicity. Microplastics can inhibit acetylcholinesterase (AChE) activity, leading to acetylcholine accumulation and subsequent disruption of cholinergic neuronal function [93], thereby inducing neurological disorders. They may also impair survival-related behaviors in fish, such as locomotion and foraging [94]. Additionally, microplastics modulate the levels of dopamine (DA), serotonin (5-HT), and γ-aminobutyric acid (GABA), thereby affecting motor control and emotion-related behaviors in fish. Exposure during the aquatic phase significantly enhanced the activities of acetylcholinesterase (AChE), cholinesterase (ChE), and choline acetyltransferase (ChAT) in the zebrafish brain in a concentration-dependent manner, whereas dietary phase exposure predominantly induced a marked increase in the concentrations of monoamine neurotransmitters dopamine (DA) and serotonin (5-HT). The combined neurotoxicity of microplastics via these dual pathways represents a significant potential risk to aquatic ecosystems [95]. Furthermore, at the molecular genetic level, microplastics can modulate the expression of neurotransmitter-related genes, thereby altering neurotransmitter abundance and consequently diminishing the adaptive capacity of fish [96]. Concurrently, microplastics induce the generation of reactive oxygen species (ROS) within cells, disrupt redox homeostasis, and inflict damage to neuronal DNA, proteins, and lipid structures, ultimately leading to oxidative stress-mediated neurotoxicity [93].

The bio-toxicity induced by microplastics is disrupting the stability of aquatic ecosystems and compromising their structural integrity [97]. Such ecological damage not only interferes with material cycling and energy flow in aquatic systems but also indirectly threatens human health and poses a potential risk to the sustainable development of fishery resources. Therefore, further in-depth research is urgently needed to clarify the underlying mechanisms and develop effective prevention and mitigation strategies.

### 4.2. Potential Risks to Human Health

Human exposure to microplastics occurs through multiple pathways, such as ingestion, inhalation, and dermal contact, inevitably leading to their entry into human tissues [98]. Furthermore, microplastics ingested by aquatic organisms, as discussed previously, can enter the human body directly or indirectly via the food chain [99], forming an “environment–organism–human” transmission chain. Research indicates that via ingestion pathways such as drinking water, alcoholic beverages, and food consumption, the conversion of varying plastic particle sizes into mass equivalents suggests a global average human ingestion rate of 0.1–5 g of microplastics per week. It is noteworthy that the actual environmental abundance of small-sized microplastics may be underestimated due to methodological discrepancies in sampling and analytical techniques [100].

**Figure 3:**
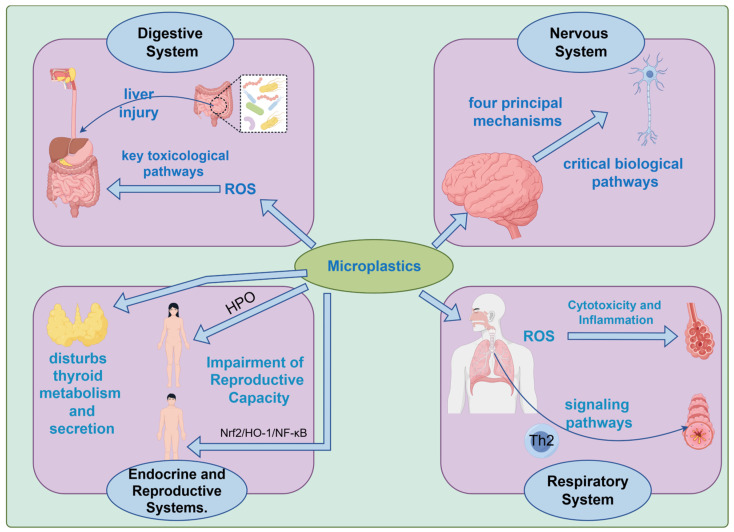
Potential Risks of Microplastics on Human Physiological Systems.

Once inside the body, microplastics can circulate via the bloodstream and distribute across tissues, interfering with and damaging physiological functions in multiple organ systems. As shown in Figure 3, the health risks are summarized below by system:

**Digestive System:** For the digestive system, it has been discovered that experimental evidence from in vitro studies and findings from animal research indicate that microplastics induce excessive generation of reactive oxygen species (ROS), serving as a molecular initiation event that triggers oxidative stress, cellular apoptosis, inflammatory responses, microbial dysbiosis, and metabolic disturbances. These key toxicological pathways collectively contribute to multifaceted and cascading damage to the two core organs of the digestive system—the intestines and liver—ultimately increasing the incidence and mortality of human digestive diseases [101]. For instance, exposure to microplastics disrupts intestinal homeostasis and compromises colonic epithelial equilibrium, thereby exacerbating colitis [102]. Moreover, the altered gut bacteria exacerbate cyclophosphamide-induced liver injury [103], amplifying the systemic toxic effects of microplastics. Although microplastics have been detected in human samples, there remains a paucity of evidence substantiating their impact on human health [101].

**Nervous System:** Animal experimentation has revealed that microplastics and nanoplastics can cross the blood–brain barrier (BBB) and elicit extensive neurotoxic effects on the nervous system [104]. Furthermore, a 2025 review indicates that microplastics contribute to neuronal stress and the progression of neurodegenerative and cognitive impairments through four principal mechanisms—direct physical damage, chemical toxicity, immune-mediated inflammation, and cellular dysfunction—as well as via critical biological pathways such as mitochondrial injury, oxidative stress, autophagosome disruption, and non-BBB penetration routes [105].

**Endocrine and Reproductive Systems:** Long-term exposure to micro- or nanoplastics in rats disturbs thyroid metabolism and secretion [106], affecting core physiological processes such as metabolism, growth, and development. Additionally, micro- and nanoplastics can disrupt gonadal hormone regulation, leading to functional and structural changes in the gonads [107]. Studies in male gonadal research have demonstrated that microplastic exposure induces abnormalities in sperm quality, histopathological damage in testicular tissue, and spermatogenic cell apoptosis in ICR male mice. The reproductive toxicity is closely associated with dysregulation of the Nrf2/HO-1/NF-κB signaling pathway. An Imbalance in this pathway triggers inflammatory responses in testicular tissue, further inducing cellular apoptosis and ultimately impairing male spermatogenic function [108]. In female organisms, microplastics can accumulate in the ovaries, uterus, and even the placenta. They directly target ovarian granulosa cells, disrupt the hormonal regulatory circuit of the hypothalamic-pituitary-ovarian (HPO) axis, impair follicular maturation and ovulation, and subsequently reduce reproductive capacity [109].

**Respiratory System:** In vitro studies utilizing human cell lines revealed that microplastics can induce cytotoxicity and inflammatory damage in alveolar epithelial BEAS-2B cells by promoting the generation of reactive oxygen species (ROS). The degradation of the pulmonary epithelial barrier occurs due to the depletion of zonula occludens-1 (ZO-1) protein. High concentrations of polystyrene microplastics (PS-MPs) are prone to trigger adverse reactions such as chronic obstructive pulmonary disease (COPD), while low concentrations compromise the pulmonary protective barrier and elevate the risk of pulmonary pathologies [110]. Concurrently, in the context of allergic airways, microplastics can activate a range of signaling pathways, including the TRPA1-p38 MAPK signaling pathway, CXCL1 signaling pathway, PI3K/AKT/mTOR signaling cascade, and NFκB signaling pathway. These signaling pathways may induce systemic sensitization and exacerbate oxidative stress, disrupt the Th1/Th2 immune balance by skewing immune responses toward a Th2 phenotype, thereby positioning microplastics as potential environmental risk factors for allergic airway diseases such as allergic rhinitis and asthma [111].

Although current direct epidemiological evidence remains scarce, multiple experimental studies suggest the potential health hazards posed by microplastics. These toxic effects are characterized by their persistence, cumulative nature, and multi-systemic impact. Particular attention must be given to susceptible groups, including pregnant women, children, the elderly, and those who are immunocompromised, a concern underscored by the detection of microplastics in human amniotic fluid [112]. Concurrently, microplastics in the natural environment can act as carriers to adsorb chemical pollutants such as heavy metals, as well as pathogens including Helicobacter pylori and SARS-CoV-2, thereby exacerbating their associated health risks to humans [113]. Helicobacter pylori can adhere to polyethylene microplastics and form biofilms, which decompose into smaller particles under the cultivation of artificial gastric fluid, thereby facilitating bacterial infiltration into the gastric mucosa and inducing tissue damage and inflammation [114]. A study utilizing SARS-CoV-2 pseudovirus (SC2-P) revealed that the virus adheres strongly to spherical polystyrene microplastics with diameters ranging from 0.5 to 2 micrometers, resulting in enhanced infectivity [115]. Interestingly, nanoscale microplastics may potentially inhibit pathogenic invasion; the underlying mechanisms and their consequential impacts necessitate further in-depth investigation [113].

Given the current scarcity of direct epidemiological evidence, future research should establish a clearer research roadmap linking environmental exposure to human health outcomes. First, systematic exposure assessment is needed to quantify microplastic intake across major pathways, including drinking water, food, and inhalation of airborne particles, while accounting for particle size distribution and polymer composition. Second, the development of reliable biomonitoring approaches is essential to evaluate internal exposure levels in humans, such as the detection of microplastics in blood, stool, placenta, and lung tissues. Standardized analytical protocols and exposure biomarkers would greatly improve the comparability of biomonitoring studies. Finally, causal inference between microplastic exposure and health outcomes should be strengthened through well-designed epidemiological studies, including prospective cohort studies and longitudinal monitoring of exposed populations. Integrating environmental exposure data, biomonitoring indicators, and health outcomes will be critical for establishing robust exposure–response relationships and improving risk assessment for microplastics.

## 5. Prevention and Control Measures

The production and use of plastics must be regulated. A multi-level governance framework for plastic pollution—spanning international, regional, national, and local scales—has been established worldwide [116]. In recent years, controls on plastic bags and microplastics have achieved notable reductions, and both enterprises and the public are increasingly participating in plastic-reduction initiatives. However, a review published in 2025 suggests that the existing international legal framework suffers from overlapping jurisdictions and fragmentation, proposing an “umbrella convention” (i.e., a Global Plastics Treaty, GPT) as a way forward [117]. Establishing a coherent global regulatory system for marine plastic pollution is therefore a critical step toward addressing microplastic contamination. Additionally, leveraging media to raise public awareness, strengthening environmental education for children and adolescents, and promoting proactive behavioral changes are essential [118]. Accelerating the link between scientific research and policy-making, and building a comprehensive plastic-reduction system involving government, industry, and the public, are also vital.

Reducing the release of microplastics is a crucial part of pollution control. The retrofitting of tertiary treatment systems has emerged as a pivotal strategy for upgrading wastewater treatment plants, significantly reducing the discharge of microplastics. Prominent approaches include established surface and depth filtration technologies, such as dissolved air flotation, oxidation ditches, micro-strainers, rapid sand filters, fabric-media filters, and MBRs [119]. Among these tertiary treatment technologies, their performance and operational characteristics differ considerably.

Dissolved air flotation technology primarily removes contaminants through the mechanisms of bubble–particle adhesion and subsequent flotation-based separation [120]. Recent research demonstrates that dissolved air flotation technology, when augmented with ferrate assistance, achieves removal rates exceeding 99% for low-to-high-density microplastics such as PE, PET, and PVC. However, proteinaceous pollutants significantly impede this removal process through competitive adsorption [121]. Disk filters can achieve an approximately 89.7% removal rate, which is influenced by the mesh aperture size. The removal efficiency is further affected by factors such as mesh deformation, rupture, or the oriented passage of particles [122]. The removal efficiency of microplastics by sand filters is contingent upon the porosity and pore size of the filtration medium, with higher retention rates observed for larger microplastic particles. The removal rate for microplastics with a diameter of 10 microns exceeds 80% [123]. However, contaminants trapped between the filter media granules and on their surfaces during the treatment process can also lead to increased retention of microplastics, necessitating periodic backwashing for such filtration systems [124,125]. Although MBRs require membrane cleaning and fouling control, increasing both construction and operational costs, their microplastic removal rate can reach 99.9%, outperforming other conventional methods [27]. Hence, operational parameters such as energy consumption levels and maintenance requirements will directly influence the overall applicability of these technologies in large-scale wastewater treatment plants.

It is noteworthy that the concept of “removal” in wastewater treatment research generally refers to the reduction in detectable microplastic particles in treated effluent. Such detection typically employs spectroscopic identification methods such as FTIR or Raman spectroscopy, which play a critical role in the quantitative analysis of microplastics [126]. Furthermore, studies indicate that FTIR significantly underestimates the quantity of microplastic particles, particularly those smaller than 50 μm, which may be overlooked [127]. Therefore, this apparent removal does not equate to the complete degradation of microplastics within the treatment system.

The sludge generated during the wastewater treatment process, which entraps microplastics, serves as a major repository for these contaminants [128]. Therefore, wastewater treatment plants can function both as barriers to microplastic retention and as pivotal nodes for their redistribution. A comprehensive review highlights the urgent need to advance sophisticated sludge treatment and resource recovery technologies in order to mitigate the environmental risks posed by microplastics [129].

The retrieval of discarded fishing nets addresses the issue of lost or abandoned fishing gear. Through grinding and lamination methods, end-of-life fishing nets can be recycled, thereby mitigating plastic pollution in the fisheries sector [130]. Promoting the use of biodegradable plastics, such as those derived from algae and other non-edible biomass, offers a potential pathway for mitigating marine microplastic pollution and building a sustainable ecosystem [131].

Intercepting microplastics during their transport phase can effectively cut off diffusion pathways, substantially reducing cross-environmental migration and accumulation in water and soil. It constitutes a pivotal component within the microplastic pollution prevention and control framework, characterized by its operational feasibility and high efficacy. Stormwater tanks, widely used for urban stormwater runoff from roads and highways [132], have been shown to retain an average of 88% of microplastics and 95% of tire-wear particles, proving effective in removing these contaminants [133]. Interception strategies include engineering-based measures such as dam interception [134] and ecological measures such as mangrove interception [135]. Constructed wetlands, commonly employed in wastewater treatment, also demonstrate high removal efficiencies: studies report microplastic removal rates of 81.63% in surface-flow constructed wetlands and 100% in horizontal subsurface-flow constructed wetlands, highlighting their effectiveness in microplastic management [136].

However, the efficiency of these interception systems in precipitating and retaining microplastics is subject to the influence of factors such as wetland vegetation root systems and substrate characteristics, flow velocity, and dam proximity [134,137]. In addition, reported removal efficiencies may vary depending on local climatic conditions, rainfall intensity, and influent characteristics such as particle size distribution and polymer composition [138,139]. Therefore, while stormwater tanks and constructed wetlands show considerable potential for microplastic interception, their performance may not be universally transferable and should be evaluated within site-specific environmental and operational contexts.

Degradation of microplastics represents a core solution, encompassing both abiotic and biotic pathways. Abiotic degradation includes photo-, thermal-, and mechanical degradation [140]. In biodegradation, the use of microorganisms as tools for breaking down microplastics has become a research focus; the process is closely linked to microbial enzymatic reactions [141]. This method has a relatively small impact on ecosystems, and future integration of biodegradation with other treatment approaches appears both feasible and promising [142].

However, the ecological implications of biodegradation processes still require careful evaluation. A review in 2024 indicated that biodegradable plastics do not fully degrade in the natural environment. Instead, they may be more prone to forming microplastics and even nanoplastics than traditional plastics, exerting similar or even stronger toxic effects on ecosystems and organisms [143]. Assessing the toxicity of these degradation products is of paramount importance, and biodegradability alone should not be considered a sufficient criterion for safety [144]. Therefore, systematic monitoring of degradation products, particle size evolution, and potential ecotoxicological impacts is necessary to ensure that biodegradation strategies do not unintentionally introduce new environmental risks.

Several targeted policy instruments have already demonstrated measurable outcomes in reducing specific sources of microplastics. For instance, regulatory bans on plastic microbeads in personal care products implemented by countries such as the United States, Canada, and the European Union have effectively eliminated a major source of primary microplastics in cosmetic formulations [145,146]. Meanwhile, a study has shown that restrictions can indeed reduce the entry of microplastics into water bodies [147]. Microfibres from textiles are also increasingly coming under regulatory scrutiny, with suggestions being made for labeling different synthetic garments, while some regions have already introduced requirements for washing machine filtration systems [148]. In the field of aquaculture and fisheries, Fishing-for-Litter (FFL) serves as an effective countermeasure, facilitating the reintroduction of captured marine debris into commercial markets or its conversion into energy [149]. Despite regional variations in the quantitative impact of these measures, the implementation of control policies targeting pollution sources can substantially reduce the emissions of microplastics.

In summary, regarding the tertiary treatment system for wastewater, although certain technologies can achieve exceptionally high “apparent removal rates,” they still face complex challenges in practical applications, including technical limitations, operational costs, and issues of pollutant transfer. Interception technologies are significantly influenced by site conditions, rainfall patterns, flow rates, and particle size distribution. The environmental safety of degradation technologies remains uncertain. In terms of policy control, greater reliance should be placed on source reduction rather than on the treatment of microplastics. Moving forward, it is imperative to integrate technological innovation, regulatory frameworks, and public participation to establish a comprehensive, synergistic prevention and control system. This represents one of the core directions in microplastic pollution research.

## 6. Conclusions and Perspectives

In recent years, the production and use of plastic products have continued to increase. Microplastics are now widely distributed in various natural water bodies worldwide, spanning freshwater, marine, polar, and deep-sea environments, with environmental concentrations rising annually. This trend poses risks to food webs and ecosystem integrity, and—through trophic transfer or environmental exposure—also constitutes a non-negligible threat to both aquatic ecosystems and human health. Research on aquatic microplastics has coalesced into four core domains: environmental transport and fate, marine ecological impacts and governance, toxicity and biological effects, and material properties and degradation. Moreover, nanoplastics and the prevention of microplastics have emerged as new research hotspots. While a multi-level regulatory framework for microplastic pollution has been established globally, a coherent and comprehensive global governance system remains to be developed. This review further emphasizes that microplastic pollution should be understood within an integrated freshwater–estuarine–marine continuum, where transport, retention, trapping, and remobilisation processes jointly regulate their environmental fate, rather than being treated as isolated system-specific phenomena. Despite significant advancements in identifying microplastic sources, environmental distribution, and removal technologies, critical knowledge gaps persist in areas such as exposure assessment, environmental fate, and effective governance strategies. In particular, inconsistencies in sampling methods, analytical techniques, and reporting units continue to hinder cross-study comparability and large-scale assessments.

Future research should focus on several priority questions that can advance both scientific understanding and practical management. First, there is a need to improve the quantification and characterization of microplastic exposure across environmental compartments in the next 3 to 5 years. Establishing standardized sampling, pretreatment, and analytical protocols, especially for smaller particles including nanoplastics, will be essential for generating reliable and comparable datasets. Secondly, it is imperative to further elucidate the environmental transformation pathways and ecological impacts of microplastics, encompassing processes such as fragmentation, aging, biofilm formation on surfaces, and interactions with co-existing pollutants. Within the next decade, particular attention should be given to long-term environmental behavior and the coupling between physical transport and biogeochemical processes. Third, advances in wastewater treatment and environmental interception technologies should be evaluated through performance metrics that reflect real operational conditions. Although many technologies demonstrate high apparent removal efficiencies, their effectiveness is often constrained by operational costs, particle size limitations, and the transfer of microplastics to sludge or other environmental compartments. Fourth, source-control strategies represent a particularly promising pathway for reducing microplastic emissions. Policy interventions targeting specific sources, such as microbeads, textile fibers, and tire-wear particles, have already demonstrated measurable effectiveness, highlighting the importance of upstream mitigation. At present, there are still no technologies that can be considered widely effective for the targeted removal of microplastics that are already dispersed throughout natural environments. Therefore, the focus of technological development in the next 5 to 10 years should prioritize scalability, environmental safety, and life-cycle impacts, rather than focusing solely on removal efficiency. Finally, growing evidence suggests that the distribution and abundance of microplastics may be influenced by broader socio-environmental factors such as climate conditions, hydrological processes, and population density. Integrating environmental monitoring with spatial modeling, big data analysis, and machine learning approaches may significantly improve the prediction of pollution hotspots and support targeted management strategies.

Overall, addressing microplastic pollution requires a synergistic approach that encompasses environmental monitoring, mechanistic studies, technological innovation, and policy formulation. Such coordinated efforts will gradually curb the proliferation of microplastic contamination, safeguard the security of aquatic ecosystems and human health, and promote the establishment of a sustainable development model characterized by harmonious coexistence between humanity and nature.

## Figures and Tables

**Table 1:** Concentration and Distribution of Microplastics Detected in Freshwater Samples.

Country	Location	Estimated MP	Sample	Size	Methods	Reference
Uzbekistan	Karasuv Canal Chirchik River Sangzor River Lake Tuzkon	137–237 items/L	Surface water	0.15–5 mm	Stereomicroscopy and Raman	[38]
Nepal	Tilicho Lake	42 items/L	Surface water	5 μm–3 mm	FTIR	[39]
Bangladesh	Old Brahmaputra River	2,239,861 items/km^2^	Surface water	11 μm–5 mm	FTIR	[40]
		253.16 items/kg	Sediment			
USA	Flathead Lake	189,000 items/km^2^	Surface water	<5 mm	Stereomicroscopy and Raman	[41]
China	Yangtze River	920,000 items/km^2^	Surface water	300 μm–5 mm	FTIR	[42]
	Poyang Lake	226 items/L	Sediment and Surface water	<5 mm	Raman	[43]
	Yellow River (lower)	430 items/L (wet) 654 items/L (dry)	Surface water	<200 μm	FTIR	[44]
Germany	River Ems	154 items/L	Surface water	250 μm–5 mm	FTIR	[45]
South Africa	Vaal River	463.28 items/kg	Sediment	<5 mm	Stereomicroscope and SEM	[46]
Canada	Great Slave Lake	420 items/L	Surface water	300 μm–5 mm	FTIR	[47]
UK	Kelvin River	161–432 items/kg	Sediment	11 μm–2.8 mm	SEM-EDS	[48]

## Data Availability

No new data were created or analyzed in this study. Data sharing is not applicable to this article.
